# Emotion response disconcordance among trauma-exposed adults: the impact of alexithymia

**DOI:** 10.1017/S0033291722002586

**Published:** 2023-09

**Authors:** Andrea Putica, Meaghan L. O'Donnell, Kim L. Felmingham, Nicholas T. Van Dam

**Affiliations:** 1 Department of Psychiatry, Phoenix Australia Centre for Posttraumatic Mental Health, University of Melbourne, Parkville, VIC, Australia; 2 Melbourne School of Psychological Sciences, University of Melbourne, Parkville, VIC, Australia

**Keywords:** Alexithymia, emotion discordance, emotion processing, trauma

## Abstract

**Background:**

Emotion processing deficits have been identified as a critical transdiagnostic factor that facilitates distress after trauma exposure. Limited skills in identifying and labelling emotional states (i.e. alexithymia) may present on the more automated (less conscious) end of the spectrum of emotional awareness and clarity. Individuals with alexithymia tend to exhibit a disconcordance between subjective experience and autonomic activity (e.g. where high levels of subjective emotional intensity are associated with low physiological arousal), which may exacerbate distress. Although there is a robust link between alexithymia and trauma exposure, no work to date has explored whether alexithymia is associated with emotional response disconcordance among trauma-exposed adults.

**Method:**

Using a validated trauma script paradigm, the present study explored the impact of alexithymia on emotion response concordance [skin conductance (Galvanic Skin Response, GSR) and Total Mood Disturbance (TMD)] among 74 trauma-exposed adults recruited via a posttraumatic stress disorder (PTSD) treatment clinic and student research programme.

**Results:**

Unlike posttraumatic symptom severity, age, sex, participant type and mood (which showed no effect on emotion response concordance), alexithymia was associated with heightened emotion response disconcordance between GSR and TMD [*F*_(1, 37)_ = 8.93, *p* = 0.006], with low GSR being associated with high TMD. Observed effects of the trauma script were entirely accounted for by the interaction with alexithymia, such that those with alexithymia showed a negligible association between subjective and physiological states.

**Conclusion:**

This finding is paramount as it shows that a large proportion of trauma-exposed adults have a divergent emotion engagement profile.

One of the hallmarks of posttraumatic stress disorder (PTSD) is abnormalities in emotion processing (American Psychiatric Association, 2013). Emotion processing is a broad term that refers to a complex set of affective, behavioural and cognitive mechanisms that underlie emotions. However, the nature of emotional processing abnormalities in PTSD has been the source of some debate – this includes attenuated (e.g. emotion numbing symptomology) as well as exaggerated emotional responsivity (e.g. hyper-arousal). Psychophysiological studies have generated consistent evidence of heightened physiological reactivity to trauma cues among individuals with PTSD relative to controls (for a review, see Orr, Metzger, Miller, & Kaloupek, 2004). Studies that have included direct comparisons of physiological responses to trauma and unpleasant non-trauma-related stimuli have shown support for trauma-specificity of emotional hyper-responsiveness in PTSD (Miller & Litz, 2004).

Hyper-activity to trauma-related stimuli is explained by Emotion Processing Theory (EPT; Foa & Kozak, 1986), which underpins the development of gold-standard treatments for PTSD, such as Prolonged Exposure Therapy (Foa, Hembree, & Rothbaum, 2007). EPT is based on Lang's bio-informational theory of fear (1977, 1979) where fear is represented in memory as structures comprised of related stimulus, response and meaning elements designed to escape danger. When something in the environment matches one or more of the fear structure elements, it is activated and spreads through the network. Applying EPT to PTSD, Foa and Rothbaum (1998) proposed that the fear structure includes excessive stimulus and response elements as well as pathological meaning elements. Foa and Kozak posit that effective psychological intervention requires activating (via arousal and engagement) and modifying the pathological elements of the fear structure. Data from clinical studies have revealed common responses indicative of emotional processing. The first is greater initial physiological reactivity during fear-relevant imagery, or physiological arousal to trauma-related stimuli (Jaycox, Foa, & Morral, 1998; Wangelin & Tuerk, 2015), taken to reflect fear-memory activation. Secondly, distress in response to accessed fear structures decreases gradually (habituates) over time as the stimuli is repeatedly presented.

However, emotion processing difficulties may be compounded by limited ability in identifying and labelling emotional states. Alexithymia is a dimensional trait strongly associated with traumatic experiences and posttraumatic pathology (Frewen, Dozois, Neufeld, & Lanius, 2008; Grabe, Spitzer, & Freyberger, 2004; McCaslin et al., 2006; Putica et al., 2021). Previous work has also shown that those with alexithymia demonstrate impaired habituation to emotionally distressing stimuli (Panayiotou & Constantinou, 2017), hypothesized to result from hypertonic arousal limiting the processing of novel emotion stimuli.

Those with alexithymia exhibit chronically elevated subjective negative affect coupled with low autonomic activity (i.e. low emotion response concordance; Connelly & Denney, 2007; Eastabrook, Lanteigne, & Hollenstein, 2013; Kleiman et al., 2016; Peasley-Miklus, Panayiotou, & Vrana, 2016). Emotional concordance, also known as emotional coherence, or emotional coupling, is characterized by within-individual correlations among physiological arousal, subjective emotional components (appraisal) and behavioural responses as emotions unfold over time. Greater coherence between physiology and subjective experience may help individuals respond more effectively to emotional stimuli. Individuals with high concordance exhibit effective responses that are matched to the magnitude of stressors and challenges. In the context of fear processing, concordance between appraisal, behavioural and physiological responses is paramount for threat perception and subsequent situational adaptation (LeDoux & Pine, 2016).

## Current study

Given the importance of posttraumatic emotion processing mechanisms in PTSD presentations and treatment, exploring individual differences in emotion processing styles is paramount. Those with alexithymia have a demonstrated tendency to respond to emotional stimuli in a disconcordant manner, characterized by attenuated physiological activity coupled with heightened subjective distress. Given the strong association between alexithymia and post-trauma pathology, it is critical to discern the role of alexithymia in emotion processing, specifically emotion response disconcordance, after trauma exposure. To our knowledge, no studies to date have explored the impact of alexithymia on emotion response concordance in PTSD. The aim of this study is to ascertain if those with alexithymia exhibit an emotional disconcordance across neutral and trauma-related stimuli during a trauma script paradigm. We predicted that alexithymia would be associated with: (1) greater PTSD severity; (2) increased subjective distress across neutral and trauma-related stimuli, and (3) greater self-reported distress, coupled with reduced galvanic skin response (greater emotion response disconcordance) across both neutral and trauma-related stimuli. Finally, we predicted that the emotion response disconcordance will not be better explained by post-trauma distress severity.

## Method

### Recruitment and participants

Various recruitment strategies were employed to recruit a sample of trauma-exposed adults. Seventy-four trauma-exposed, treatment-seeking adults (50 females; *M*_age_ = 32.72 years, s.d. = 10.57; *n* = 52, 70%) and a convenience trauma-exposed student population (*n* = 22, 30%) participated in the current study. The participants of the current study identified as Caucasian-Australian (55%), Asian (30%) and European – other (15%). All participants had completed high school, with 30% completing a bachelor's degree or equivalent qualification and the remaining 15% completed graduate-level study. The socioeconomic status of the participants was distributed as follows: 20% high, 60% middle and 20% low.

To maximize sample size and breadth of post-trauma distress, a multi-sample recruitment strategy was employed: (1) via a PTSD clinic and (2) via a university Research Experience Program – where a significant proportion of students endorsed the PTSD Checklist for the DSM-5 (PCL-5) scores indicative of traumatic exposure and probable PTSD. The treatment-seeking sample was recruited from a PTSD clinic and the trauma-exposed student sample was recruited from a pool of psychology students. To be eligible, participants had to endorse a historical experience of a traumatic event as listed in the Life Events Checklist (Weathers et al., 2013a). All participants endorsed experiencing at least one traumatic event. A summary of the endorsed traumatic events used to record the trauma script is presented in the online Supplementary Information. Eighty-five per cent of the participants reported experiencing PCL-5 scores indicative of probable PTSD (total scores >33). Forty-nine per cent of participants had a Toronto Alexithymia Scale (TAS-20) score indicative of alexithymia (⩾61).

### Design

Patients seeking treatment for PTSD underwent the experimental procedure prior to treatment. For participants recruited within the treatment trial, trauma script information was gathered (as per Wangelin & Tuerk, 2015) at the clinical assessment session, with the trauma script paradigm presented at the start of the first treatment session. The average time between trauma script data collection and the experimental session for the patient population was 6 days. The average time between data collection and the experimental procedure was 20 minutes for students – with the student completing the self-report measures while the researcher audio-recorded the trauma script. All data and the experimental task took place during a single hour lab session. All research activities were granted ethics approval by local ethics committees.

#### Trauma script task data collection

Information was gathered about the participant's index traumatic experience. A standardized template (Wangelin & Tuerk, 2015) was completed eliciting the participant's trauma narrative, along with sensory probes about the experience (sights, sounds, smells, physiology). The completed template was used to prepare an audio recording of the participant's index trauma event. To facilitate standardization, each script required descriptors from at least three of the following five categories: sights, sounds, sensations, smells and tastes, and at least one thought or emotion approximately every 30 seconds during the 3 minute script. Each script was recorded by the same female researcher to minimize effects of vocal variation across script presentations. Sleep, medication and substance use (including caffeine) in the preceding 12 h period were documented on the task flow sheet which was used to ensure standardization and fidelity.

### Procedure

**Fig. 1.**
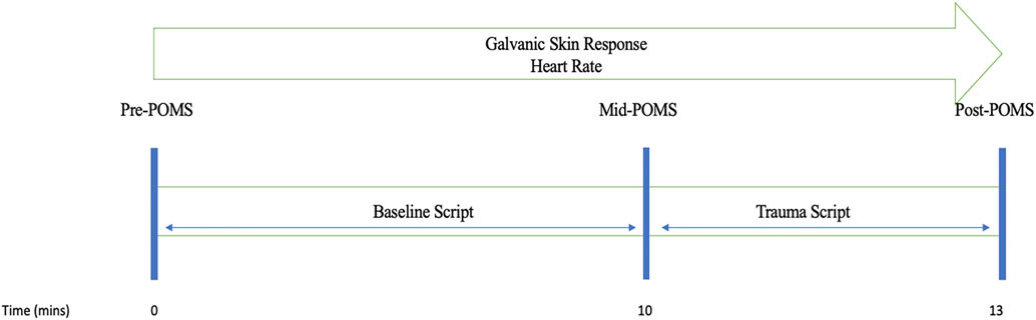
Experimental session flow.

Participants completed the Profile of Mood States (POMS) prior to the baseline script, prior to the trauma script and after the trauma script. See Fig. 1 below for a session flow diagram. Participants were asked to rate their subjective level of physiological arousal prior to commencing and at 1 minute intervals during the trauma script. The task was conducted in a private room, with the temperature maintained between 20 and 22 degrees Celsius (as per manufacturer's instructions). For the task, the participant was seated, with sensors and headphones placed by the therapist/researcher. The standardized neutral script was presented first via audio, followed by the trauma script. Participants were instructed to shut their eyes and ‘imagine, as vividly as possible, the following scene’. As used by Wangelin and Tuerk (2015), a standard neutral nature script was presented for 10 minutes to acquire neutral psychophysiological measurement. A longer neutral script duration was chosen to allow ample time for acclimation to the situation, considering that the trauma script was presented only once (as per Wangelin & Tuerk, 2015). A neutral period of 10 minutes is also recommended by the manufacturer of the physiological sensors (Mindfield Biosystems, Inc., Berlin, Germany). After hearing the neutral script, the participants were presented with a personalized 3 minutes trauma script via audio. All scripts were presented at 60 decibels through Sony MDR-ZX110NC noise-cancelling headphones.

### Measures

#### Life Events Checklist for the DSM-5

The Life Events Checklist for the DSM-5 (LEC-5; Weathers et al., 2013a) was used to screen for trauma exposure. The LEC-5 assesses exposure to 16 events known to potentially result in PTSD or distress and includes one additional item assessing any other extraordinarily stressful event.

#### PTSD Checklist for the DSM-5

PCL-5 (Weathers et al., 2013b) is a 20-item self-report measure used to assess the 20 DSM-5 symptoms of PTSD. Scores consist of a total symptom severity score ranging from 0 to 80. The scale consists of four sub-scales which are directly linked to the PTSD diagnostic symptom clusters. The PCL-5 is used to monitor PTSD symptomology, screen individuals for PTSD and make provisional PTSD diagnoses. Responses on the measure range on a 0–4 rating scale (0 = ‘not at all’ to 4 = ‘extremely’). Previous studies reported that the PCL-5 exhibited strong internal consistency (***α*** = 0.94), test-retest reliability (*r* = 0.82), convergent (*r* = 0.74–0.85) and discriminant (*r* = 0.31–0.60) validity (Blevins, Weathers, Davis, Witte, & Domino, 2015). Cronbach's *α* for our sample was *α* = 0.81 for the total PCL-5 score.

#### Toronto Alexithymia Scale

TAS-20 (Bagby, Taylor, & Parker, 1994) was used to measure the presence and severity of alexithymia. The TAS-20 is a self-report scale comprised of 20 items rated using a five-point Likert scale (1 = *strongly disagree* and 5 = *strongly agree*). There are five items that are reverse scored (items 4, 5, 10, 18 and 19). The TAS-20 contains three sub-scales: Difficulties Identifying Feelings (DIF), Difficulties Describing Feelings (DDF), and Externally Orientated Thinking (EOT). Good test-retest reliability and excellent internal consistency, with *α* coefficients ranging from 0.74 to 0.77, have been reported (Bagby et al., 1994). The total alexithymia score is the sum of responses to all 20 items, while the score for each sub-scale factor is the sum of the responses to that sub-scale. Cronbach's *α* for our sample was ***α*** = 0.83 for the total TAS-20 score.

#### Profile of Mood States

The Abbreviated POMS (Grove & Prapavessis, 1992) is a 40-item self-report measure of distinct, transient mood states used to measure subjective emotional reactivity. Items are scored on a five-point Likert scale (0 = *not at all* and 4 = *extremely*). Two items on the esteem-related affect sub-scale are reverse-scored prior to being summed with other items. The POMS was used to measure appraisals of affect before and after completing the trauma script task to assess the subjective distress at neutral, after presentation of the neutral script and as a response to the trauma script task. A *Total Mood Disturbance* (TMD) score is calculated by summing the totals for the negative sub-scales and then subtracting the totals for the positive sub-scales. Therefore, a negative score indicates a positively valenced mood. The abbreviated measure has good demonstrated reliability (*r* = 0.80) and good discriminant validity (Grove & Prapavessis, 1992). In our sample, the Cronbach's *α* for the pre-POMS was 0.96 and, for the post-POMS, was 0.97.

#### Physiological response

Mindfield eSense sensors were used to measure heart rate (HR) and Galvanic Skin Response (GSR). HR was continuously measured using eSense Pulse over the chest (polyamide and conductive silicone; electrical contacts were made of Thermoplastic Polyurethane), using a one-way channel ECG with 500 Hz, RR intervals with 5 Hz sampling which continuously transmits the time between two heartbeats to the eSense application via Bluetooth. The HR device has a frequency range of 2457 MHz with a measuring range of 20–240 BPM (±2 BPM). It was operated with 3 V DC voltage.

GSR was measured via the eSense Skin Response Sensor, at 5 Hz, by placing two Velcro electrodes on the participant's middle and index finger and connecting them to a smart device. The Skin Response Sensor used direct voltage with a voltage source of 0.61 V DC and a source resistance of 61.5 kOhm. The resolution of the measured values was 18-bit, rounded to 2 decimal places (i.e. 0.01) *μ*S in data export without rounding. The measuring range was 10 kOhms to 1 MOhm corresponding to a range of 100–1 *μ*S. Skin response data were log-transformed prior to analysis.

Data were exported as a csv file directly from the device running the respective applications at the completion of the procedure to a centralized data email address. Any deviations in task procedure or concerns regarding data integrity (including electrode concerns) were noted on the procedure sheet and used to check the data for quality. A latency window of 1 s was observed for both measurements. Prior to data analysis, data were screened for sources of error in eSense recording, including discontinuity in recording. Data with widespread or multiple excursions were excluded from the study.

### Statistical analysis approach

Statistical analysis was performed using the SPSS software package, version 27. Potential group differences were examined. Data were screened and manipulation checks were performed. Results showed significant changes in subjective responding, GSR and emotion response concordance from neutral to trauma script. Fisher's *Z*-transformations were applied to individual scores of psychophysiological and subjective affect measurements using the mean and standard deviation of each variable and the entire sample to allow comparison of variables with different levels of measurement. The variables were then used to create within-person correlation coefficients of arousal and appraisal (i.e. concordance). The main advantage of a concordance correlation coefficient is that it accounts for systemic bias and random error when assessing the concordance between two variables or a test and re-test (Lin, 1989). Levene's test and normality checks were conducted.

Physiological data were down-sampled to 5 Hz. Mean HR and GSR levels were calculated for each script (neutral, trauma). The difference in mean HR (BPM) and GSR (microsiemens; *μ*S) between neutral and trauma conditions (i.e. trauma script–neutral script) was calculated to index trauma-specific HR and GSR reactivity. Due to the difference in script length between neutral (10 minues) and trauma (3 minutes) conditions, an alternative calculation of emotion response concordance was also performed by comparing mean concordance during the first 3 minutes of the neutral script with mean concordance during the trauma script (3 minutes). No differences in results were observed when concordance was calculated in this manner (see online Supplementary Information), therefore, all analyses were reported using mean concordance for the full neutral script length in the reactivity calculation. We ran repeated-measures ANCOVAs with age, sex, alexithymia and baseline mood (where appropriate) as covariates on TMD, GSR and emotion response concordance. Significant interactions observed for alexithymia were followed up with median split (high alexithymia ⩾ 59 < low alexithymia) ANCOVAs to illustrate response patterns between those with low and high alexithymia. Nominal *α* was set at *p* < 0.05. Multilevel modelling and ANOVA analyses were conducted, given the findings were similar only ANOVA results will be reported.

## Results

### Sample data

Prior to conducting our analyses, we explored between-sample characteristics to ensure our samples were comparable (see online Supplementary Table S1). No significant differences between gender and PCL-5 scores were observed between samples (see online Supplementary Fig. S1). However, there was a significant difference between the samples in age and alexithymia, therefore analyses with age as a covariate were conducted as part of our main analyses. Alexithymia was a variable of interest and was already a planned covariate.

### Manipulation checks

A manipulation check indicated that the trauma script had its intended effect. There was a main effect of the script phases [*F*_(1.66, 58.07)_ = 34.05, *p* < 0.001], with the neutral script decreasing TMD (*d* = 0.39), the trauma script increasing TMD (*d* = 0.82) and TMD peaking during the trauma script (*d* *=* 0.50). There was also a main effect of script phases [*F*_(1, 43)_ = 20.02, *p* < 0.001], with participants showing peak GSR at the commencement of the neutral script which decreased over experimental phases (*d* *=* 0.36). However, our manipulation check did not reveal a significant change in average HR from neutral to trauma script phases [*F*_(1, 48)_ = 0.52, *p* = 0.47). Due to the null effect for HR, main analyses exploring concordance across HR measures were not conducted.

### Hypothesis: alexithymia is associated with greater PTS severity

Distributional information for all data is presented in the online Supplementary Information (Table S2). Prior to conducting our main analyses, intercorrelations of our study variables were explored (see online Supplementary Table S3). Alexithymia was found to be associated with higher PTSD symptom severity [*r*(74) = 0.51, *p* = 0.001] and subjective distress as measured by TMD scores (TMD as derived from the POMS, TMD at neutral and trauma script).

### Hypothesis: alexithymia is associated with greater subjective distress across phases

Repeated-measures ANCOVAs controlling for alexithymia, age, sex, participant type (student or treatment seeker) and baseline mood (where appropriate) were conducted to determine effects of experimental phase on TMD, GSR and concordance, as well as to examine any interaction with TAS-20 (alexithymia) scores. Regarding TMD, a significant interaction was observed between alexithymia and phase [*F*_(1, 40)_ = 4.34, *p* = 0.04]. However, there was no main effect of the script phases on TMD [*F*_(1, 40)_ = 0.16, *p* = 0.70]. No interaction effects were found for age [*F*_(1, 40)_ = 1.64, *p* = 0.21], sex [*F*_(1, 40)_ = 1.80, *p* = 0.19] or participant type [*F*_(1, 40)_ = 2.6, *p* = 0.114].

#### Impact of alexithymia on distress across phases

**Fig. 2.**
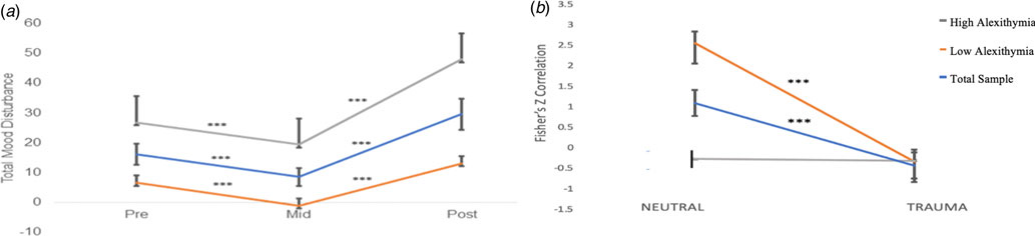
Total sample and alexithymia median split across experimental phases. (*a*) Total Mood Disturbance (TMD) scores across the pre-neutral, post-neutral and post-trauma script phases; (*b*) emotion response concordance (correlation between TMD and GSR) across the neutral and trauma script time points. ***Significant at *p* < 0.001.

To illustrate the impact of alexithymia on emotion response concordance across phases, we performed a median split of TAS-20 scores (high alexithymia ⩾ 59 < low alexithymia; see Fig. 2). We then performed repeated-measures time × TMD ANOVAs for the low and high alexithymia groups individually. A significant effect of phase was found on TMD among those with low alexithymia [TAS-20 ⩾ 59; *F*_(1, 22)_ = 20.33, *p* < 0.001] between neutral (*M* = 7.91, s.d. = 10.57) and trauma script phases (*M* = 16.74, s.d. = 15.16; Fig. 2*a*). A significant effect on phase was also found for the high alexithymia group [*F*_(1, 22)_ = 20.33, *p* < 0.001] between neutral (*M* = 26.83, s.d. = 23.60) and trauma script phases (*M* = 46.50, s.d. = 33.68; Fig. 2*a*). Although both the low and high alexithymia groups showed an increase in negative affect in the transition from neutral to trauma phases, the high alexithymia group demonstrated greater negative affect across both conditions and the greatest difference in negative affect in the transition from neutral to trauma phases.

### Hypothesis: alexithymia is associated with lower skin response across phases

Regarding GSR, there was no significant main effect for phase [*F*_(1, 31)_ = 1.52, *p* = 0.27]. No significant interactions were observed for age [*F*_(1, 31)_ = 1.95, *p* = 0.17], sex [*F*_(1, 31)_ = 2.81, *p* = 0.60], baseline mood [*F*_(1, 31)_ = 3.4, *p* = 0.09] or alexithymia [*F*_(1, 31)_ = 0.84, *p* = 0.37].

### Hypothesis: alexithymia is associated with greater emotion response disconcordance across phases

Regarding emotional concordance, there was a significant effect of alexithymia which showed a significant interaction with phase [*F*_(1, 37)_ = 8.93, *p* = 0.006]. However, there was no main effect of the script phases [*F*_(1, 37)_ = 0.17, *p* = 0.68]. No interaction effects were found for age [*F*_(1, 37)_ = 0.2, *p* = 0.88], sex [*F*_(1, 37)_ = 0.05, *p* = 0.83], participant type [*F*_(1, 37)_ = 0.18, *p* = 0.68] or baseline mood [*F*_(1, 37)_ = 0.29, *p* = 0.60].

#### Impact of alexithymia on response concordance across phases

To investigate the interaction with alexithymia, we performed a median split by alexithymia scores (high alexithymia ⩾ 59 < low alexithymia). Those with low alexithymia demonstrated significant change in concordance from neutral (*M* = 2.57, s.d. = 1.68) to trauma script [*M* = −0.32, s.d. = 2.95; *t*_(34)_ = 5.29, *p* < 0.001]. However, no significant difference in response concordance was found for the high alexithymia group from neutral (*M* = −0.21, s.d. = 2.51) to trauma script [*M* = −0.51, s.d. = 2.51; *t*(_28)_ = 0.67, *p* = 0.50; see Fig. 2*b*]. The lack of effect for phase on concordance for the high alexithymia group suggests that these individuals have a consistent or anticipatory disconcordant response style, regardless of emotional demand or context. On the contrary, a significant effect of phase was found [*F*_(1, 34)_ = 27.99, *p* < 0.001, *d* = 1.20], for those in the low alexithymia group between neutral (*M* = 2.57, s.d. = 1.70) and trauma script phase (*M* = −0.32, s.d. = 2.95; Fig. 2*b*) on emotion response concordance. These differential effect findings among the low alexithymia group suggest that those low in alexithymia only show disconcordant responding during the trauma script phase, demonstrating that they have differential concordance between experimental contexts. For concordance correlations across experimental phases, see online Supplementary Materials (Fig. S2).

### Hypothesis: emotion response disconcordance is not accounted for by PTS severity

To ensure that overall sample interaction between alexithymia and emotion response concordance across experimental phases was not influenced by PTSD symptom severity we conducted a follow-up rmANCOVA treating PTSD severity as covariates for the whole sample, respectively. We did not find PTSD severity [*F*_(1, 72)_ = 3.71, *p* = 0.60] to be a significant covariate for emotion response concordance across phases.

## Discussion

Adaptive emotion processing is theorized to be imperative in protecting individuals from the development of psychopathology. Specifically, work to date suggests that those with greater emotion response concordance may be better able to meet the transitional, context-specific demands, and in turn experience greater wellbeing (Levenson, 2014; Luhmann, Hofmann, Eid, & Lucas, 2012). The current study explored the impact of alexithymia on emotion reactivity concordance during neutral and trauma script conditions among trauma-exposed adults. Our study showed that alexithymia was associated with greater posttraumatic distress severity, subjective distress across all experimental conditions (neutral and trauma script) and emotion response disconcordance. Our findings add to the established literature demonstrating that alexithymia is associated with chronic emotion processing deficits (i.e. emotion disengagement) regardless of emotional demand. Specifically, we found the most profound emotion response disconcordance among those with alexithymia during both the neutral and trauma script conditions. Our findings were not accounted for by posttraumatic distress severity, baseline mood, sex or age, supporting previous work showing that alexithymia is a related but separate construct to posttraumatic distress (Putica et al., 2021).

The emotion response disconcordance observed as a function of alexithymia is characterized by lower physiological arousal (GSR) while endorsing heightened subjective negative affect during the neutral phase. However, we did not find a differentiation in emotion response disconcordance between neutral and trauma script conditions for those in the alexithymia group, with the group exhibiting disconcordance across both phases. The observed disconcordance is consistent with previous findings demonstrating that those with alexithymia exhibit a comparable response regardless of emotional demand (Connelly & Denney, 2007; Eastabrook et al., 2013; Kleiman et al., 2016; Peasley-Miklus et al., 2016). The lack of differentiation in concordance between phases is consistent with the suppression hypothesis – where it is believed that those with alexithymia overuse suppression as an emotion regulation strategy resulting in a paradoxical experiencing of emotional distress/intensity coupled with lower correlations between emotional responses (i.e. physiology, behaviour and appraisal; Butler, Gross, & Barnard, 2014) and threat expectancy (Hennings, Bibb, Lewis-Peacock, & Dunsmoor, 2021). A lack of observed differentiation in emotion response concordance may be due to individuals with alexithymia reaching their peak emotion response disconcordance during neutral phases (anticipation of impending emotion), limiting resources for adaptive modulation to acute emotional demands (i.e. presentation of trauma memory). High neutral emotion response disconcordance may be due to hypertonic arousal, which in turn blunts an individual's adaptive phasic arousal (i.e. ability to respond to novel stimuli). The dysregulated tonic and phasic arousal hypothesis may also explain previous findings that those with alexithymia show impaired habituation to emotionally distressing stimuli (Panayiotou & Constantinou, 2017). Our work suggests that alexithymia may be a predisposing factor for posttraumatic emotion response disconcordance, which may impact an individual's ability to engage in fear extinction, increasing the likelihood of posttraumatic psychopathology and treatment resistance.

### Clinical implications

Our findings have tentative clinical implications. The findings of disconcordant responding would suggest that extinction-based therapies, which require high levels of arousal activation coupled with modulating distress, may not be useful for those with high levels of alexithymia. As front-line treatments for PTSD tend to be fear extinction-based, levels of alexithymia may present as a barrier for treatment response and may contribute to poor treatment response to current treatments.

### Limitations and future research

To our knowledge, this is the first study to explore emotion response concordance in trauma-exposed individuals. While our findings show that there was a correlation between alexithymia and PTSD symptom severity, future research would benefit from comparing disconcordance between trauma-exposed controls and those with PTSD to identify if this is a disorder-specific phenomenon. We recognize that participant self-selection and using convenience sampling may have biased our data. Similarly, our use of self-report measures may be a further limitation, as asking those who have difficulty with emotional awareness to report on their levels of emotional awareness is inherently problematic. Finally, the sample in the current study was relatively homogenous, further work across more diverse samples is warranted. It is also important to note that although the task employed in the current study was one of emotion processing, given that the relationship between emotion processing and regulation is complex and dynamic, with people processing stimuli in the context of goals, our task may have also been capturing mechanisms of emotion regulation. Future work should incorporate measures of emotion regulation with concordance tasks. Despite this limitation, our work does highlight a potential mechanism of treatment non-response observed for trauma-focused PTSD treatments. Our findings could be further strengthened by future work exploring how emotion response disconcordance impacts PTSD treatment engagement and outcomes.

## Conclusion

Emotion processing deficits have been identified as a critical transdiagnostic factor that facilitates distress after trauma exposure. The current study shows that unlike trauma symptom severity, age, sex or mood (which showed no effect), individuals with alexithymia may present with a unique emotion response style – disconcordance between subjective experience and autonomic activity (i.e. low emotion response concordance). Future work is needed to examine how alexithymia and emotion response disconcordance may impact treatment outcomes for trauma-focused treatments.

## Supplementary material

For supplementary material accompanying this paper visit https://doi.org/10.1017/S0033291722002586. click here to view supplementary material
