# Supplementary material for: Emotion response disconcordance among trauma-exposed adults: the impact of alexithymia
Source: Psychol Med. 2022 Aug 17;53(12):5442–8. doi: 10.1017/S0033291722002586 (PMC10482720; doi:10.1017/S0033291722002586)
Supplement: Supplementary file 1 [file S0033291722002586sup001.docx]

**Supplementary**

**Alternative Calculation of Concordance**

When we compared the mean concordance between the first three minutes of the neutral script with the three-minute trauma script, a significant interaction between alexithymia and phase was found, *F*(1, 38) = 4.09, *p =* .05. No main effect was found for phase, *F*(1, 38) = 0.28, *p* = .60. Further, sex, *F*(1, 38) = 0.40, *p* = .53, age, *F*(1, 38) = 1.25, *p* = .27, and baseline mood, *F*(1, 38) = 0.244, *p* = .624, were not found to be covariates in this alternate model.

**Participant Trauma Exposure**

Table S-1

*Lifetime Traumatic Events Endorsed by Participants*

| Event | Frequency |
| --- | --- |
| Natural disaster | 3 |
| Transportation accident | 6 |
| Serious accident at work, home, or during recreational activity | 1 |
| Exposure to toxic substance | 1 |
| Physical assault | 12 |
| Assault with a weapon | 4 |
| Sexual assault | 9 |
| Other unwanted or uncomfortable sexual experience | 16 |
| Captivity | 1 |
| Life-threatening illness or injury | 8 |
| Severe human suffering | 1 |
| Sudden violent death | 3 |
| Sudden accidental death | 6 |
| Serious injury, harm, or death you caused to someone else | 3 |

Table S-2

*Descriptive Statistics*

| Modality | Measures | Mean *(SD)* | Range |
| --- | --- | --- | --- |
| Self-Report |  |  |  |
|  | TAS-20 | 59.70 (2.33) | 33.00 - 85.00 |
|  | PCL-5 | 38.67 (16.55) | 4.00 – 78.00 |
|  | B-TMD | 17.95 (20.61) | -11.00 – 80.00 |
|  | T-TMD | 32.09 (29.73) |  |
| Physiological |  |  |  |
|  | B-GSR | 2.33 (2.49) | 0.69 – 14.20 |
|  | T-GSR | 3.43 (3.52) | 0.52 – 14.80 |
|  | B-HR | 80.38 (15.30) | 54.60 – 115.00 |
|  | T-HR | 81.39 (18.66) | 15.2 – 122.00 |
| Concordance |  |  |  |
|  | B-GSR | 2.57 (1.68) | -1.49 – 6.67 |
|  | T-GSR | 1.18 (1.42) | -6.06 – 5.26 |
|  | B-HR | 2.65 (3.00) | 2.21 – 3.09 |
|  | T-HR | 0.38 (3.00) | -5.82 – 6.18 |

Table S-3

*Total Score Intercorrelations Table*

|  | 1 | 2 | 3 | 4 | 5 | 6 | 7 | 8 | 9 | 10 |
| --- | --- | --- | --- | --- | --- | --- | --- | --- | --- | --- |
| 1.Age | - |  |  |  |  |  |  |  |  |  |
| 2. Sex | .33** | - |  |  |  |  |  |  |  |  |
| 3.TAS-20 | .06 | .-32 | - |  |  |  |  |  |  |  |
| 4. PCL-5 | -.14 | -.17 | .51* | - |  |  |  |  |  |  |
| 5. BGSR | -.25 | .18 | - .12 | .22 | - |  |  |  |  |  |
| 6. TE-GSR | .22 | .06 | -.10 | .01 | -.29 | - |  |  |  |  |
| 7. BHR | -.01 | -.35* | .11 | -.01 | .02 | .80 | - |  |  |  |
| 8. TE-HR | .25 | .17 | .04 | -.41* | .35 | -.19 | .13 | - |  |  |
| 9. BTMD | .22 | -.24 | .53** | .64** | -.19 | -.01 | .19 | -.09 | - |  |
| 10. TE-TMD | .33* | -.10 | .57** | .67** | -.21 | -.03 | .13 | .00 | .93 | - |

*Note.* TAS-20 = Toronto Alexithymia Scale; PCL-5 = PTSD Checklist for DSM-5; BGSR = Average Neutral Galvanic Skin Response; TE-GSR = Average Galvanic Skin Response; BHR = Average Neutral Heart Rate; TE-HR = Average Trauma script Heart Rate; BTMD = Total Neutral Mood Disturbance; TE-TMD = Total Trauma script Mood Disturbance.

* *p* < .05.

** *p* < .001.

Table S-4

*Between Treatment-Seeking (n = 52) and Student Sample (n = 22) Comparisons*

|  | Students  Mean (*SD*) or *Frequency | Patients  Mean *(SD*) or *Frequency | Between-group differences |
| --- | --- | --- | --- |
| Age | 21.5 (5.21) | 37.43 (8.86) | *t*(67) = 7.81, *p* = .001 |
| Sex (F) | 15* | 31* | X^2^(1) = 0.03, *p* = .85 |
| TAS-20 | 62.47 (11.70) | 54.36 (13.06) | *t*(67) = 2.48, *p =* .02 |
| PCL-5 | 39.30 (11.15) | 40.31 (18.72) | *t*(67) = 0.25, *p* = .80 |

*Note.* F = Females; TAS-20 = Toronto Alexithymia Scale-20 PCL-5 = PTSD checklist for the Diagnostic and Statistical Manual of Mental Disorders.

**Between-Samples Manipulation Checks**

***Student Sample.*** A manipulation check suggested that the trauma script had its intended effect. There was a main effect for script phase, *F*(2, 36) = 19.01, *p* < .001, *d* = 0.52, with the neutral script reducing TMD (*M* = 2.58, *SD* = 14.55, *d = 0.45*), while the trauma script increased TMD (*M* = 19.53, *SD* = 21.81, *d* = 0.56; Figure S-1. Secondly, a main effect for phase was observed for GSR, *F*(1, 15) = 27.71, *p* = .001, *d* = 0.83, with higher values from neutral (*M* = 3.10, *SD* = 2.26) to trauma script (*M* = 5.67, *SD* = 3.76; Figure S-1. A further expected main effect for phase was observed for GSR-TMS concordance, *F*(1, 21) = 7.04, *p* = .02, *d* = 0.69, with greater concordance observed during the neutral script (*M* = 1.33, *SD* = 2.20), which reduced during the trauma script phase (*M* = -0.40, *SD* = 2.78; Figure S-1.

***Patient Sample.*** A manipulation check suggested that the trauma script had its intended effect. There was a main effect for script phase, *F*(2, 32) = 17.15, *p* < .001, with the neutral script reducing TMD (*M* = 15.18, *SD* = 19.42, *d* = 0.38), while the trauma script increased TMD (*M* = 40.76, *SD* = 36.77, *d* = 0.56; Figure S-1. Secondly, a main effect for phase was observed for GSR, *F*(1, 22) = 12.50, *p* = .002, *d* = 0.10, with higher observed GSR during the trauma script (*M* = 2.31, *SD* = 2.95) compared to the neutral condition (*M* = 2.01, *SD* = 2.81; Figure S-1). However, no main effect for phase was observed for GSR-TMS concordance, *F*(1, 46) = 6.90, *p* = .12 (Figure S-1).

*
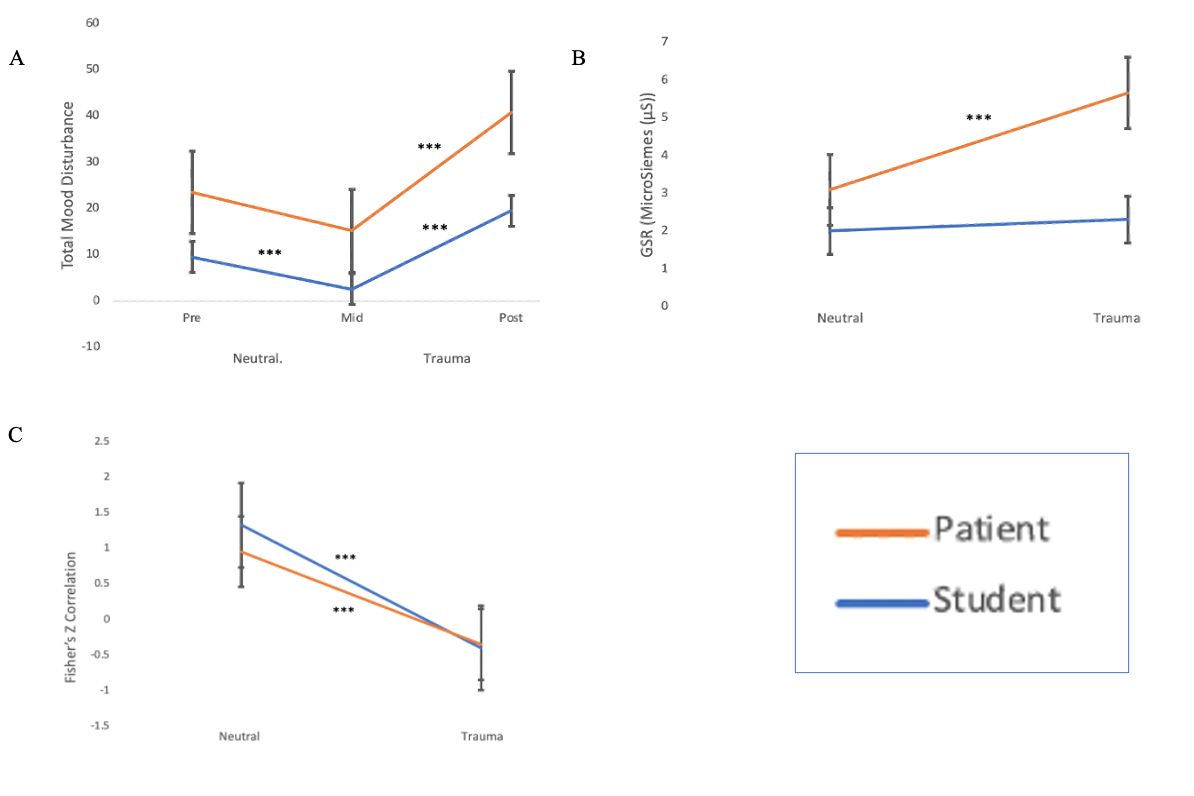
*

*Figure S-1.* Between-Samples Outcomes Across Experimental Phases. (A) Patient and student samples TMD changes across experimental phases; (B) Patient and student samples GSR changes across experimental phases; (C) Patient and student samples emotion response concordance changes across experimental phases.

*** Significant at p < .001.


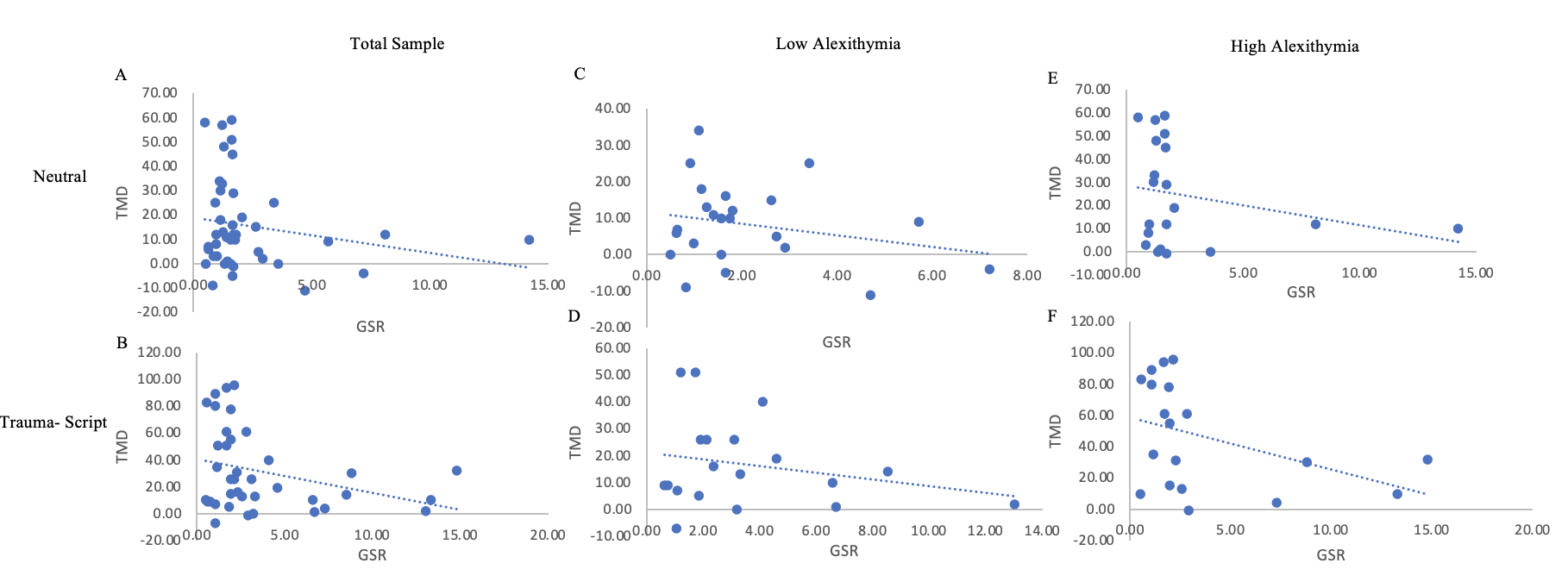


*Figure S-2.* Concordance Correlations Across Experimental Phases. (A) Total sample TMD and GSR correlations during neutral phase; (B) Total sample TMD and GSR correlations during trauma script phase; (C) Low Alexithymia TMD and GSR correlations during neutral phase; (D) Low Alexithymia TMD and GSR correlations during trauma script phase; (E) High Alexithymia TMD and GSR correlations during neutral phase; (F) High Alexithymia TMD and GSR correlations during trauma script phase.
